# Supplementary material for: Wave-Particle Duality Relation with a Quantum Which-Path Detector
Source: Entropy (Basel). 2021 Jan 18;23(1):122. doi: 10.3390/e23010122 (PMC7831905; doi:10.3390/e23010122)
Supplement: Supplementary file 1 [file entropy-23-00122-s001.pdf]

Supplementary material

"Wave-particle duality relation with a quantum which-path detector"

by Dongyang Wang, Junjie Wu, Jiangfang Ding, Yingwen Liu, Anqi Huang, and Xuejun Yang

In this document, we exhibit the measured coincidence counts which are used to calculated the quantum coherence C and path distinguishability D.

I. COINCIDENCE COUNTS FOR THE MEASUREMENT OF C

We derived the quantum coherence C from the density matrix ρ_q of the quanton photon, which is obtained by the single-qubit tomography (QST) on it. Since for most of the cases, the quanton photon is entangled with the QWPD (i.e. quantum which-path detector) photon, ρ_q can be represented as follows,

$$\rho_q = p_H \rho_{qH} + p_V \rho_{qV}, \tag{1}$$

where p_H is the probability that the QWPD photon is found to be in the state of $|H\rangle_d$, and ρ_{qH} is the density matrix of the quanton photon under such a circumstance. p_V and ρ_{qV} have the similar definitions for $|V\rangle_d$.

In experiment, we first set the polarization projection on the QWPD photon as $|H\rangle\langle H|$, and then recorded the six kinds of coincidence counts when the polarization projection on the quanton photon was set as $|H\rangle\langle H|$, $|V\rangle\langle V|$, $|D\rangle\langle D|$, $|A\rangle\langle A|$, $|R\rangle\langle R|$ and $|L\rangle\langle L|$, respectively, where $|D\rangle = \frac{1}{\sqrt{2}}(|H\rangle + |V\rangle)$, $|A\rangle = \frac{1}{\sqrt{2}}(|H\rangle - |V\rangle)$, $|R\rangle = \frac{1}{\sqrt{2}}(|H\rangle + i|V\rangle)$, and $|L\rangle = \frac{1}{\sqrt{2}}(|H\rangle - i|V\rangle)$. These counts are denoted as n_{HH}, n_{HV}, n_{HD}, n_{HA}, n_{HR} and n_{HL}, respectively. ρ_{qH} was calculated from these six counts by QST. The same procedures were repeated when the polarization projection on the QWPD photon is $|V\rangle\langle V|$, and we recorded counts as n_{VH}, n_{VV}, n_{VD}, n_{VA}, n_{VR} and n_{VL}, respectively, and obtained ρ_{qV}. p_H and p_V were derived from

$$p_H = \frac{n_{HH} + n_{HV}}{n_{HH} + n_{HV} + n_{VH} + n_{VV}}, \quad p_V = 1 - p_H. \tag{2}$$

Finally, ρ_q was obtained from Eq.(1), and C was calculated as twice the absolute value of ρ_q's off-diagonal element.

Average of the coincidence counts are listed in Table.S1, which were recorded per 0.5 second. When $\alpha = \frac{\pi}{2}(90°)$, since the QPWD photon is in the state of $|H\rangle_q$ (i.e. $p_H = 1$), only the first six kinds of coincidence counts were required.

TABLE S1. Average of the coincidence counts for the measurement of C

α	n_{HH}	n_{HV}	n_{HD}	n_{HA}	n_{HR}	n_{HL}	n_{VH}	n_{VV}	n_{VD}	n_{VA}	n_{VR}	n_{VL}
0°	12.8	156.6	54.3	72.1	57.1	59.0	107.6	9.8	50.0	66.7	51.3	67.2
10°	12.8	171.5	61.9	123.1	102.8	75.8	109.2	8.1	60.4	67.9	61.0	67.7
20°	17.3	180.3	48.1	152.8	110.8	84.4	106.7	7.4	59.3	66.0	58.3	60.9
30°	23.4	170.4	35.3	134.8	105.5	78.3	100.9	6.5	54.0	56.0	61.0	54.1
40°	35.0	169.9	24.6	184.0	116.0	78.0	85.7	5.4	40.8	55.4	51.2	45.2
50°	42.3	154.2	16.6	194.5	107.3	71.3	70.8	4.9	31.2	44.2	47.2	29.0
60°	51.5	126.0	6.8	180.0	106.0	61.3	44.5	4.0	18.0	34.1	28.5	24.8
70°	59.6	103.5	3.2	174.6	98.0	47.1	28.7	3.9	14.8	20.7	16.1	19.5
80°	69.3	81.5	2.1	161.0	64.7	79.6	14.0	4.6	4.5	11.4	8.4	8.3
90°	72.6	65.4	4.5	145.1	59.4	63.4						
100°	38.0	54.8	1.6	90.4	41.3	48.1	14.1	3.8	8.8	10.3	9.9	9.1
110°	43.0	29.3	5.4	69.3	35.7	32.8	7.8	6.0	11.3	3.5	4.9	8.7
120°	39.6	17.3	20.8	33.9	40.5	11.6	23.3	7.0	21.3	10.7	8.6	23.7
125°	22.2	2.4	14.3	15.5	9.0	6.9	28.2	3.8	16.7	14.2	15.4	14.8
130°	31.1	40.1	32.3	52.1	65.1	3.9	50.5	7.1	33.8	29.0	13.1	45.1
140°	10.9	13.7	21.0	2.4	7.5	10.4	51.7	3.8	29.7	29.6	13.0	9.9
150°	10.6	20.2	26.6	3.6	11.1	12.7	64.6	3.7	27.8	29.8	19.3	24.3
160°	20.8	86.8	53.6	50.3	78.2	12.4	117.2	7.4	71.8	62.4	36.1	83.7
170°	12.7	94.7	55.6	55.3	80.3	22.4	123.8	6.8	70.5	69.4	36.7	87.7
180°	8.4	115.8	54.5	69.9	60.3	49.8	132.0	6.4	75.3	75.3	64.0	67.2

II. COINCIDENCE COUNTS FOR THE MEASUREMENT OF D

In this section, we list in Table.S2 the coincidence counts of n_{H0}, n_{H1}, n_{V0}, and n_{V1}, which correspond to Eq.(31) in the main text. The experiment settings are the same as those used for the measurement of C.

TABLE S2. Average of the coincidence counts for the measurement of D

α	n_{H0}	n_{H1}	n_{V0}	n_{V1}
0°	11.6	121.1	116.0	8.7
10°	10.5	129.5	124.5	9.3
20°	8.5	123.2	131.5	9.0
30°	8.9	116.4	127.7	7.9
40°	11.1	102.9	126.1	10.2
50°	10.7	88.9	105.2	11.1
60°	13.5	63.9	56.4	11.3
70°	17.7	43.3	65.9	12.4
80°	24.8	27.6	43.2	14.1
90°	15.2	22.1	19.2	20.4
100°	9.2	50.8	12.5	17.4
110°	7.3	47.1	10.6	12.8
120°	3.8	31.6	10.8	4.2
125°	6.7	58.3	11.7	7.3
130°	4.1	54.2	16.0	8.9
140°	7.1	45.3	20.6	6.3
150°	6.4	56.4	53.9	8.7
160°	9.8	83.8	80.4	7.6
170°	8.2	91.3	94.0	5.4
180°	8.6	114.0	119.8	5.7
